# Integrated Microfluidic Lectin Barcode Platform for High-Performance Focused Glycomic Profiling

**DOI:** 10.1038/srep20297

**Published:** 2016-02-02

**Authors:** Yuqin Shang, Yun Zeng, Yong Zeng

**Affiliations:** 1 Department of Chemistry, University of Kansas, Lawrence, KS 66045; 2 College of Water Resource and Hydropower, Sichuan Agricultural University, Ya’an, Sichuan 625014, P.R. China; 3 University of Kansas Cancer Center, Kansas City, KS 66160.

## Abstract

Protein glycosylation is one of the key processes that play essential roles in biological functions and dysfunctions. However, progress in glycomics has considerably lagged behind genomics and proteomics, due in part to the enormous challenges in analysis of glycans. Here we present a new integrated and automated microfluidic lectin barcode platform to substantially improve the performance of lectin array for focused glycomic profiling. The chip design and flow control were optimized to promote the lectin-glycan binding kinetics and speed of lectin microarray. Moreover, we established an on-chip lectin assay which employs a very simple blocking method to effectively suppress the undesired background due to lectin binding of antibodies. Using this technology, we demonstrated focused differential profiling of tissue-specific glycosylation changes of a biomarker, CA125 protein purified from ovarian cancer cell line and different tissues from ovarian cancer patients in a fast, reproducible, and high-throughput fashion. Highly sensitive CA125 detection was also demonstrated with a detection limit much lower than the clinical cutoff value for cancer diagnosis. This microfluidic platform holds the potential to integrate with sample preparation functions to construct a fully integrated “sample-to-answer” microsystem for focused differential glycomic analysis. Thus, our technology should present a powerful tool in support of rapid advance in glycobiology and glyco-biomarker development.

Protein glycosylation is probably the most common post-translational modification. It is estimated that up to 50% of human plasma proteins are glycosylated, including disease biomarkers that originate from impaired cells and tumor tissues^1^. Despite the biomedical significance, progress in glycomics has considerably lagged behind genomics and proteomics^2^. Protein glycosylation is challenging to analyze owing to its dynamic and heterogeneous nature caused by the non-templated biosynthesis^3^. Human plasma proteins span a dynamic concentration range of ~10 orders of magnitude and glycoproteins of interest, such as cancer biomarkers, are often present at very low levels^2^,^4^, which makes it extremely difficult to accurately measure their glycan changes. Systems glycomic profiling is further complicated by the structural diversity of human glycome, which is estimated to contain more than 10^3^–10^4^ oligosaccharide species^5^. At present, mass spectrometry (MS) is a powerful technology for structural analysis of glycoproteins and has been the gold standard method in glycomics. However, MS-based glycan analysis usually requires large sample volume and multi-step sample preparation^6^,^7^. Such tedious and time-consuming process compromises quantification accuracy and substantially limits throughput for large-scale clinical studies to correlate glycosylation aberrations with the physiological and pathological status. In recently years, lectin microarray has emerged as a useful platform that complements MS-based methods for glycomic studies^8^,^9^. Lectin microarray offers a simple, rapid and high-throughput tool for probing specific populations of glycoprotein motifs, extensively profiling of lectin-glycan interactions, and the whole tissue-level studies of human plasma glycome to identify disease-specific glycan signatures^10^,^11^. A commonly used format of lectin array employs surface patterned lectins to capture glycoproteins that are pre-labeled for direct fluorescence detection. While this method has been valuable for glycomic profiling of complex samples or pre-purified glycoproteins, it lacks the ability for sensitive and quantitative measurements, due in part to the need of fluorescent labeling of samples^8^. To complement this method, sandwich types of lectin array assisted by antibodies have been developed for the study of glycosylation of specific proteins^11^,^12^. Kuno *et al*. reported the antibody-overlay lectin array (abbreviated as “antibody-lectin array” hereafter) in which specific antibodies were used to detect the target glycoproteins captured by the lectins on the surface^12^. This method can use the same antibodies for enrichment and expression analysis of targeted glycoproteins, which not only enables profiling of glycosylation changes on disease-specific or tissue-specific biomarkers, but also greatly increases the sensitivity, specificity and reproducibility than the direct detection method that requires pre-labeling of samples. Nonetheless, these lectin-based assays suffer from an intrinsic limitation due to the weak lectin-glycan interactions with affinity constants K_a_ = 10^4^–10^7^ M^−1^ in comparison to K_a_ = 10^8^–10^12^ M^−1^ for antibody-antigen interactions^13^,^14^. To enhance the sensitivity and specificity, rigorous sample processing (e.g., immunodepletion and separation) and extremely long incubation times for lectin-glycan assay (usually overnight) are required^11^,^13^. Thus, it is imperative to develop new lectin microarray technologies that confer substantially improved sensitivity, speed and reproducibility.

Microfluidics has shown unique advantages for bioanalysis. It allows us not only to promote reaction kinetics to substantially improve analysis sensitivity and speed, but also to achieve unprecedented analysis throughput while reducing sample consumption through large-scale integration^15^,^16^,^17^. While microfluidics has made tremendous impact on genomics and proteomics, very limited progress in glycomics has been reported. Most efforts have been focused on miniaturization of glycan separation^18^,^19^ and coupling with MS detection^20^,^21^. A lectin blotting microsystem has been recently demonstrated, which has limited scalability for multiplexed glycan analysis due to the serial gel photopatterning method used for lectin immobilization^22^. To our best knowledge, only one microfluidics-based lectin array system has been reported, which encloses a surface-patterned lectin microarray insides a single channel^23^. However, this system still used the direct fluorescence detection method^8^,^14^, which requires fluorescent labeling of glycoproteins purified from clinical samples.

To address the aforementioned issues, we have developed an integrated and automated microfluidic lectin barcode-platform to substantially improve the performance of the antibody-lectin microarray. Based on this platform, we developed an on-chip sandwich antibody-lectin barcode assay and a very simple blocking method to effectively suppress the background noise. Using this system, specific glyco-profiling and sensitive detection of two standard *N*-linked glycoproteins were achieved with a substantially improved sensitivity and speed than conventional lectin arrays. To demonstrate the feasibility of our system for focused differential glycomic profiling, we measured tissue-dependent glycosylation changes of an ovarian cancer biomarker CA125 from various sources in a high-throughput fashion. Highly sensitive detection of CA125 was also achieved with a detection limit much lower than the clinical cutoff value for cancer diagnosis. With the high sensitivity and specificity, our technology could provide a powerful focused glycomic profiling platform to detect dynamic glycan changes of glycoproteins of interest, for instance, recombinant therapeutic proteins and disease biomarkers. The prototype device integrates eight parallel channels each with an array of 16 lectins, which allows for parallel measurements of up to eight glycoproteins. The multiplicity and throughput of the microfluidic system can be readily scaled up to achieve the systems glycomic analysis of a large panel of biomarkers specific to disease or tissues to explore their unique glycosylation patterns. Thus, our technology should provide a useful tool that complements the existing lectin array technologies to facilitate the elucidation of complex human glycome and the development of glyco-biomarkers.

## Results

### Microfluidic Design and Operation

**Figure 1:**
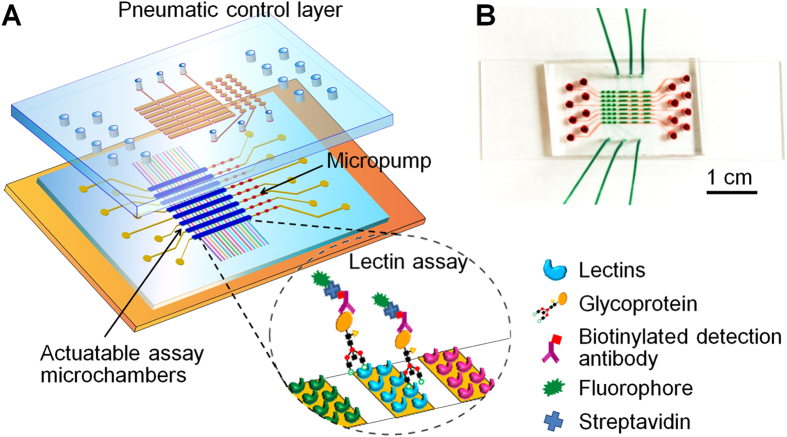
Multi-channel microfluidic lectin barcode assay system. (**A**) Schematic illustration of the chip design and the scheme of antibody-lectin sandwich assay (inset). The two-layer PDMS chip integrates eight parallel units each consisting of a three-valve pump and an actuatable assay chamber. The assembled chip is sealed onto a glass slide with the assay chambers aligned vertically across an array of lectins patterned on the substrate surface. Glycoproteins are captured by the arrayed lectins and detected by biotinylated antibodies and fluorescently labeled streptavidin. Pneumatic actuation of the assay chamber promotes fluidic mixing for fast affinity binding. (**B**) Digital photo of an assembled chip filled with red food dye in the bottom flow channels and green dye in the pneumatic control channels.

Figure 1 shows the prototype microfluidic lectin barcode chip that we have devised. The three-layer device integrates eight parallel units to demonstrate the scalability of the system. Fluidic channels were fabricated on a ~200 μm thick PDMS membrane sandwiched by a glass substrate and a PDMS slab containing the pneumatic control circuit. Each unit integrates a three-valve micropump and a lectin micro-barcode assay microchamber that can be pneumatically actuated. A lectin barcode array was patterned on the substrate surface as the sensing elements for the antibody-lectin assay involving lectin capture of glycoproteins and fluorescence detection using biotinylated antibodies and dye-labeled streptavidin (Fig. 1A).

Sensor geometries such as channel height and sensor size have been found to be the key parameters that affect binding kinetics in channel flow^24^,^25^,^26^,^27^. We tested the microfluidic lectin barcode assay with a fixed channel height of 30 μm and varying lectin bar width from 50 to 250 μm. The 100-μm bar was found to confer optimal combination of sensitivity and array density. To gain insights into the sensing behavior of the device, we analyzed a set of dimensionless parameters following the convection-reaction-diffusion (CRD) theory which provides a convenient means to qualitatively assess the mass transport regimes and binding kinetics of surface-based sensing systems^24^. For simplicity, we assessed RNase B–ConA binding under steady laminar flow as the model system. Typical geometric and kinetic parameters used are: RNase B concentration *c*_0_ = 10 nM; averaged flow rate *Q* = 0.2 μL min^−1^; chamber width *W*_c_ = 300 μm; surface density of immobilized ConA *b*_m_ = 10^12^ cm^−2^ 24,26; diffusion coefficient of RNase *D* = 1.2 × 10^−6^ cm^2^ s^−1^; association constant (*K*_a_) and dissociation rate constant (*k*_d_) on the order of 3 × 10^6^ M^−1^ s^−1^ and 1.5 × 10^−3^ s^−1^, respectively^13^,^28^. The Peclet numbers that describe mass transport scaled to the channel height and lectin bar width were first computed to be Pe_H_ = 92.5 and Pe_S_ = 6050, respectively. The large Pe values (Pe ≪ 1) indicate that flow convection dominates analyte transport to the lectin barcode, forming a thinner analyte depletion zone (thickness δ ≈ 5.5 μm) than the channel height and the lectin bars. Thus our sensing system is operated in the diffusion-limited regime to facilitate the binding kinetics in comparison to the convection-limited systems^29^. Under this mass-transport condition, the Damkohler number (Da) was calculated by Da = *k*_a_*b*_m_δ/D = 4.2 × 10^−3^, suggesting the equilibration of sensors fall in the reaction-limited regime (Da ≪ 1) rather than the diffusion-limited regime (Da ≪ 1). The system is estimated to equilibrate on a characteristic reaction time scale *t*_R_ = (*k*_d_ + *k*_a_ *c*_0_)^−1^ ≈ 11 min, which predicts the theoretical speed limit that glycan-lectin affinity dictates. These results verify that our microfluidic design can drive the lectin assay towards the reaction-limited kinetics to improve the analytical performance in comparison to the conventional formats that are usually mass transport-limited^25^,^30^.

**Figure 2:**
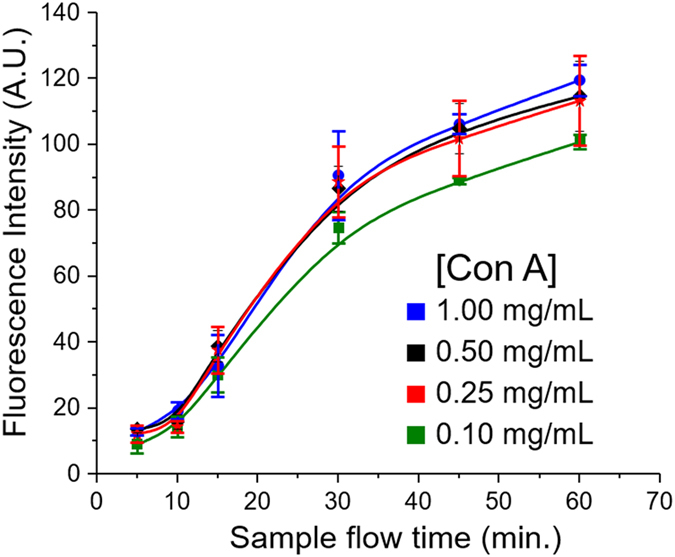
Lectin binding of glycoprotein in the microfluidic system. A mannose-binding lectin, Con A, was patterned on the chip surface at various concentrations. RNase B samples (5 μg/mL) were pumped through the channels at an averaged flow rate of ~0.2 μL/min for varying period of time.

In addition, integrated pneumatic pumps and valves allow us to actively control the flow delivery and mass transport. Here a five-step stop-flow pumping method that we developed^31^ was adapted with slight modification: the on-chip pump was operated at 0.25 Hz with four consecutive valve actuation steps set to 0.5 s each and a pulse step of 2 s. The flow rate can be readily controlled by adjusting the closing pressure while holding the opening vacuum at −87 kPa for valve actuation^32^. The optimal volumetric flow rate was determined to be ~0.2 μL/min generated at 55 kPa closing pressure, which is consistent with our previous study^31^ with the geometries factored in and the flow rate range predicted by numerical simulation^30^. In addition to controlling flow delivery, the valves aligned on the top of each assay chamber were actuated briefly to generate convective mixing to facilitate affinity binding^33^,^34^,^35^. Using these flow conditions, the platform was characterized by detecting RNase B pumped through the channels for various incubation time with ConA (Fig. 2). Higher ConA concentrations for patterning result in faster binding rate and better signal until signal saturation occurs above 0.25 mg/mL. The experimental characteristic reaction time was determined to be ~30 min, which is on the same order of the time scale theoretically predicted above. Given the relatively strong affinity of RNase B to ConA (10^−7^ M), the incubation time for our lectin assays were normally extended to two hours, which is much faster than traditional lectin microarrays usually requiring overnight incubation^8^,^14^.

### Microfluidic Sandwich Antibody-Lectin Barcode Array

**Figure 3:**
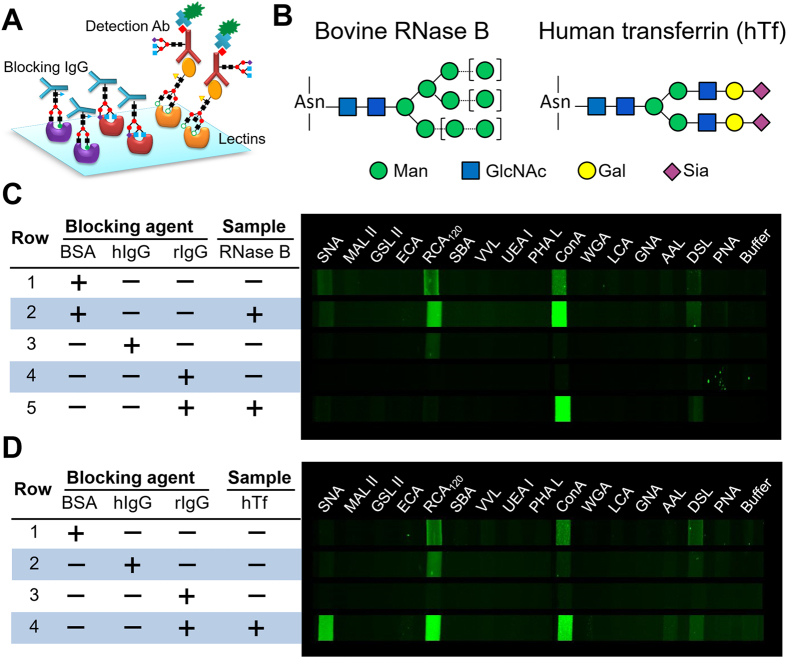
Development and characterization of the microfluidic antibody-lectin sandwich barcode assay. (**A**) Binding of the glycosylated detection antibody on the arrayed lectins can cause severe non-specific background. To mitigate this effect, the target-bound lectin array is blocked with a non-labeled IgG blocker prior to detection using a biotinylated antibody and fluorescently labeled streptavidin. (**B**) The predominant glycan structures on the standard glycoproteins used: bovine RNase B and human transferrin (hTf). The square brackets indicate the possible high mannose variants on RNase B. (**C**,**D**) False-color fluorescence images of glyco-profiling of bovine RNase B (**C**) and hTf (**D**) using a 16-lectin barcode array and different blockers-BSA, human plasma IgG (hIgG) and rabbit serum IgG (rIgG). The concentrations of RNase B and hTf are 5 and 50 μg/mL, respectively.

The performance of the antibody-lectin array is largely interfered by various noise sources: physical adsorption, non-specific lectin-glycan interactions and more significantly, binding of glycosylated detection antibodies on unoccupied lectins (Fig. 3A). To investigate these effects, a panel of 16 lectins was selected to confer binding specificities to the major glycan groups (Table S1). The standard glycoproteins that we chose are RNase B with high mannose structures and human transferrin (hTf) which is mainly modified with GlcNAc, tri-mannose, Galβ1-4GlcNAc (LacNAc) and terminal α2-3–linked sialic acid (Fig. 3B)^36^. We first studied the surface blocking and buffer conditions to minimize the background due to physical adsorption and non-specific lectin-glycan interactions. A benchtop lectin assay in the microplate format was implemented where glycoproteins were biotinylated for fluorescence detection to avoid the interference of glycosylated antibodies (Figure S1). This simple assay allowed us to conveniently optimize a variety of experimental variables, including running buffers, concentrations of divalent metal ions, and blocking reagents. It was found that the Carbo-Free™ blocking solution can effectively reduce the background levels and non-specific lectin responses without inhibition on lectin-glycan binding, contrary to another common blocking buffer tested (Figures S2 and S3). Quantitative glycoprotein detection was achieved using various lectins with good specificity to glycan structures (Figure S4 and Table S1). These data not only greatly facilitated the development of the new microfluidic sandwich lectin barcode array, but also served as a “gold standard” for technology validation, as delineated below.

Lectin binding of glycosylated detection antibody presents a major cross-reactivity issue to the antibody-lectin assay. A rational blocking strategy is to use a non-labeled IgG carrying the same or even more glycan variants than those of the detection antibody (Fig. 3A). We first studied the IgGs from the same host species as that of the detection antibody, i.e., rabbit immunoglobulins (rIgG), for blocking, as presented in Fig. 3C,D. Relatively high analyte concentrations were deliberately used to observe very weak interactions. When BSA was used for blocking (Fig. 3C, row 1 and 2; Fig. 3D, row 1), background signals were mainly observed on RCA120, ConA, DSL and SNA which possess specificities for galactose and LacNAc, complex-type *N*-glycans, (GlcNAc)_n_, and Neu5Acα2-6Gal(NAc) epitopes, respectively. These background signals recognize a general pattern of IgG Fc *N*-glycans composed of predominantly core-fucosylated, biantennary complex-type structures, some of which may carry a α2–6 sialic acid residue on the antenna^37^. It is also noted that the RCA120 background for both rabbit antibodies was higher than that of SNA, which is the opposite of the pattern reported previously using a rabbit polyclonal anti-hTf antibody^12^. Fairly strong undesired binding of antibodies with RCA120 was also observed using the lectin-antibody sandwich assay^11^. These different lectin-binding patterns reflect well documented variations in galactosylation and sialylation of mammalian IgG *N*-glycans^37^,^38^. The performance of blocking with human plasma IgG (hIgG) was then evaluated, as first described by Kuno *et al*.^12^. While being able to suppress the majority of non-specific signals in the absence of both glycoproteins, hIgG was ineffective to block the interactions between the rabbit detection antibodies and RCA120 (Fig. 3C,D), suggesting the structural difference in galactose containing *N*-glycans between the rabbit antibodies and hIgG blocker.

**Figure 4:**
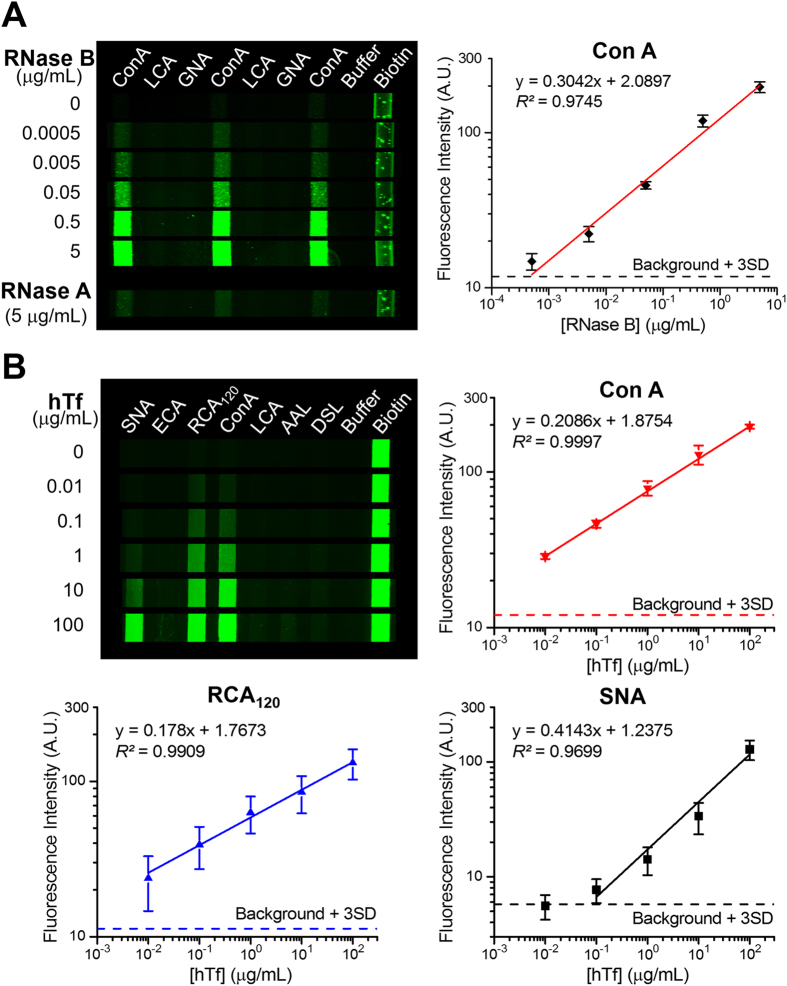
Specific and quantitative glycoprotein detection using the microfluidic lectin barcode array. (**A**) Representative fluorescence images (left) and log-log calibration plot (right) of detecting RNase B by ConA in comparison to non-glycosylated RNase A. (**B**) Fluorescence images and log-log calibration curves for detection of hTf using an array of seven lectins. Error bars are standard deviations (S.D., *n* = 3) and the dashed lines indicate the background level plus three S.D. measured with the blank for individual lectins.

The IgG-based blocking method can be much improved by using the detection antibodies and blocking IgGs from the same host species. This simple method resulted in substantially reduced non-specific signals, especially on RCA120 (Fig. 3C, row 4; Fig. 3D, row 3). As expected, detection of RNase B yielded intense signal only on ConA which selectively binds α-Man^39^. The observed lectin binding profile of hTf is consistent with the previous reports^11^,^40^,^41^, e.g., relatively strong SNA signal from the Siaα2-6Gal/GalNAc moieties and weak AAL signal for the Fucα1-6GlcNAc group that was occasionally present on hTf^36^,^41^. In contrast to SNA, another sialic acid-binder, MAL II lectin, shows no binding signal due to its preferable affinity to the α2-3 linked sialic acid, indicating the feasibility of choosing proper lectin combinations for specific determination of glycan structural variations. Some other blocking reagents that we have tested, such as a mixture of hIgG and disaccharide, may block the lectin barcode array well, but also inhibit specific glycan-lectin binding (Figure S5). Titration experiments of RNase B display very low background for the blank and two non-responding lectins and a linear response on ConA over a 10^5^ dynamic range (Fig. 4A), with a limit of detection (LOD) calculated to be 0.45 ng/mL (30 pM). Comparative analysis of RNase glycoforms shows a signal close to the background level for non-glycosylated RNase A at 5 μg/mL. Quantitative analysis of hTf was carried out using seven lectins selected according to the profiling results of hTf (Fig. 4B). ConA, RCA120, and SNA were observed to confer more sensitive detection of hTf, yielding a LOD of 0.16, 0.096 and 65 ng/mL, respectively. The lectin binding behavior observed by the microfluidic assay was verified by the conventional microplate lectin assays (Figure S4). These studies should have demonstrated that rIgG is effective in blocking non-specific binding in the antibody-lectin assay, while preserving specific signals of the targets.

### Focused Differential Glycan Profiling

**Figure 5:**
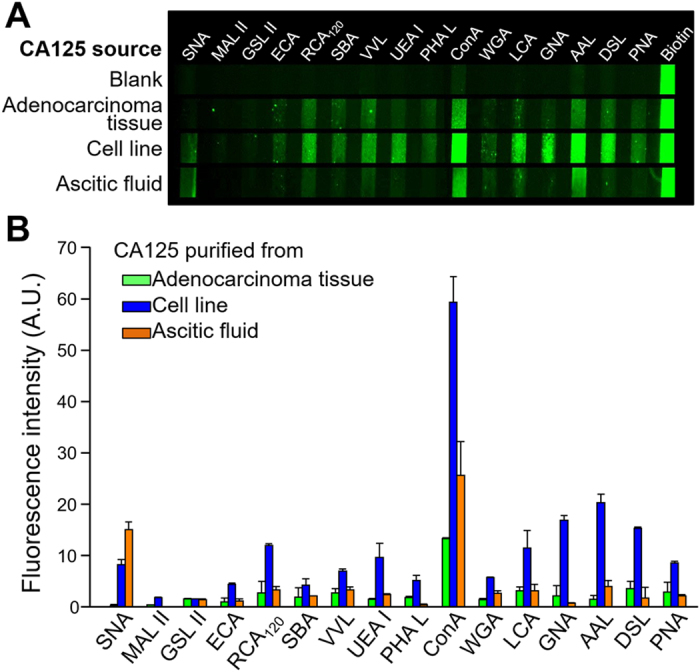
Focused differential glycomic profiling of protein biomarker using the microfluidic lectin barcode array system. (**A**) Representative fluorescence images showing different lectin binding profiles for native CA125 proteins purified from human adenocarcinoma, ovarian carcinoma cell line, and human ascitic fluids, respectively. (**B**) Background-corrected bar graph for quantitative comparison of the lectin binding profiles of different CA125 proteins demonstrates the ability to identify unique glycomic patterns of disease markers. The concentrations of all CA125 samples were 7500 units/mL. Error bars indicate S.D. (*n* = 2).

To demonstrate the feasibility for high-performance focused differential profiling of disease-associated glycan aberrations, we performed a proof-of-concept study of a well-studied ovarian cancer marker, CA125, using a mouse anti-human CA125 mAb for detection. The blank test in Fig. 5A showed very low non-specific lectin binding of the mouse antibody which can be attributed to the more heterogeneous glycan composition of rIgG than mouse IgG^42^. Figure 5 summarizes the glycoprofiling results of three human CA125 protein samples purified from ovarian cancer cell line and adenocarcinoma tissue and ascetic fluids from ovarian cancer patients (Table S2). A common lectin binding feature was observed. First, consistent strong binding with the *N*-glycan binder, Con A, indicates the predominant expression of high mannose and/or complex bisected type (bi-, tri-, and tetra-antennary) glycans. The presence of tri-, and tetra-antennary complex *N*-glycans was further verified by binding with more specific lectins PHA-L and DSL^43^. Three CA125 samples bound consistently to WGA, RCA120, VVL, SBA and PNA. WGA and RCA120 signals suggest the presence of polylactosamine type *N*-glycans, which was confirmed by DSL, and *O*-linked glycans, such as Galβ1-4GlcNAc on the core 2 type glycans^44^. VVL has reactivity for terminal GalNAc in various glycans including the simplest *O*-linked monosaccharide GalNAcα-Ser/Thr (i.e., Tn antigen), while SBA confers better specificity to Tn antigen and GalNAcGalβ1-3GalNAcα-Ser/Thr (core 2 *O*-glycan) and PNA detects Galβ1-3GalNAcα-Ser/Thr (core 1 or T antigen)^45^. Both *N*- and *O*-linked glycans on CA125 are mostly fucosylated, which was recognized here by LCA and AAL for the core-fucosylation and UEA specifically for outer arm-fucosylation^45^,^46^. Compared to the non-cancerous conditions, sialylation level of CA125 in ovarian cancer was found to be increased with a shift from α2-3 to α2–6 linkage^45^,^47^,^48^. As seen in Fig. 5, such subtle structural feature was detected by the differential binding responses of two sialic acid-reactive lectins: relatively high binding with SNA (specific for Neu5Acα2-6Gal/GalNAc) and weak binding with MAL-II (specific for Neu5Acα2-3Galβ1-4GlcNAc/Glc). Collectively, the lectin binding profile obtained here is in general agreement with the *N*- and *O*-glycans of CA125 from OVCAR3 cell line, human amniotic fluid, placenta, and serum^43^,^44^,^45^,^46^,^48^,^49^, which were revealed using a lectin-overlay-antibody array^50^, lectin-based assay^46^ and affinity chromatography^45^,^48^, and MS^44^,^46^.

Considerable difference in these CA125 lectin binding profiles was also observed with the largest discrepancy between the cell line-derived CA125 (cl-CA125) and the other two: adenocarcinoma tissue (at-CA125) and ascites fluid-derived CA125 (af-CA125). Most lectins reacting with CA125 showed stronger signal to cl-CA125 than at- and af-CA125 except SNA, suggesting higher degree of CA125 glycosylation in the cultured cells. More specifically, these significantly elevated glycans include: high mannose (detected by ConA and GNA) and tri-/tetra-antennary complex (PHA-L and DSL) *N*-glycans; the *O*-glycans with Core 1 (PNA), Core 2 (RCA120) and Tn antigen (VVL) structures; and both α1-2 linked (UEA) and α1–6 linked fucose (AAL) at outer and core positions. The sialic acid specific lectin SNA showed a distinct binding pattern from other glycans with the highest binding with af-CA125 and almost no signal for at-CA125. This finding indicates the source-sensitive variation in the extent and/or the structure of CA125 sialylation, which might provide rich information about the pathological conditions of cancer patients^50^.

**Figure 6:**
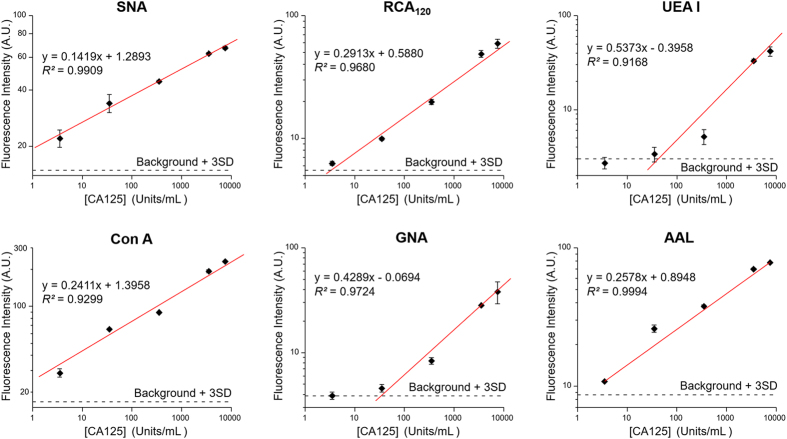
Evaluation of quantitative detection of CA125. The lectins were selected to target various glycan structures on CA125 obtained from the ovarian cancer cell line according to the glycan profiling results shown in Fig. 5. The calibration curves were obtained by the least-squares fitting. Error bars indicate S.D. (*n* = 3).

To further characterize the microfluidic platform for quantitative glyco-profiling of CA125, detection of cl-CA125 from 3.5 to 7500 U/mL were calibrated using six selected lectins (Fig. 6). These lectins showed varying sensitivity and LOD targeting different carbohydrate motifs of cl-CA125, which are in agreement with their relative binding strength observed in Fig. 5. The LODs obtained for individual lectins were calculated to be 0.153 U/mL for SNA, 3.30 U/mL for RCA120, 42.0 U/mL for UEA I, 0.188 U/mL for ConA, 33.8 U/mL for GNA, and 1.44 U/mL for AAL. The LODs of some of the lectins, such as SNA, ConA and AAL, are much lower than the current clinical cutoff value (35 U/mL) for diagnosis of ovarian cancer^51^. In particular, these lectins of high sensitivity are of significant clinical relevance because aberrant alterations in *N*-glycans, fucosylation, and sialylation have been implicated in ovarian tumors^43^,^45^,^50^,^52^.

## Discussion

Compared to many biological interactions, glycan-lectin affinity is intrinsically weak, underscoring the importance of controlling reaction kinetics to improve the performance of lectin-based glycomic assays. Our microfluidic platform was designed to address two key elements affecting surface-based sensing: sensor geometries and fluid transport. The assessment based on the CRD theory verifies that our microfluidic design can drive the lectin assay towards the reaction-limited kinetics to greatly improve the analytical performance and speed in comparison to the conventional formats that are usually mass transport-limited^25^,^30^. Furthermore, unlike the existing microfluidic lectin array that relies on passive diffusion mixing in constant laminar flow^26^, our pneumatic valve-based microfluidic architecture allows for implementation of a ‘stop-flow’ method for fluid transport. Previous experimental and theoretical studies have shown that the stop-flow method enables more efficient surface capture of targets than the continuous flow process, especially with slow binding kinetics, low concentrations or small sensing features^31^,^53^. Thus our microfluidic system is able to substantially enhance the speed of lectin-based assay for high-throughput glycomic profiling (Fig. 2). The easy-to-integrate valve and pump structures hold the potential for full automation of the microsystem.

Lectin array has emerged as a powerful tool for high-throughput glycomic profiling. Three major variants of lectin array have been developed and in this study we systematically investigated the antibody-lectin assay first introduced by Kuno *et al*.^12^. Compared to the most widely used direct detection format^8^,^14^, this strategy eliminates the need to fluorescently label purified glycoproteins which can pose challenges to clinical applications. Another type of sandwich assay uses an immobilized antibody array to capture glycoproteins, followed by detection using fluorescently labeled lectins^11^. However, this approach involves chemical modification of glycans on the capture antibodies to mitigate non-specific background. While being able to probe a large number of glycoproteins, this array technique detects one glycan by one lectin at a time, necessitating repetitive and lengthy assays to explore complex glycomic profiles. In contrast, the antibody-lectin configuration enables highly multiplexed analysis of glycan structures on a glycoprotein of interest and can be readily combined with the sample preparation steps such as immunoprecipitation and enrichment for clinical analysis^8^,^9^,^12^.

However, a major issue of the antibody-lectin assay arises from undesired lectin binding of glycosylated detection antibodies. Blocking with hIgG has been reported^12^, which is simple and has no adverse effects on the activity of antibodies compared to the methodology that chemically modifies the antibody glycans^11^. However, our results show that this approach has limited blocking effectiveness to different detection antibodies (Fig. 3), suggesting source-dependent glycan structures of antibodies. Raju *et al*. reported a comprehensive MALDI-TOF-MS study of IgGs produced by 13 animal species, which revealed species-specific variations in IgG glycosylation, especially in terminal galactosylation and sialylation of IgGs^42^. These results indicate that glycosylation variations of IgGs significantly affect the non-specific background of the antibody-lectin assay and the blocking effectiveness of IgGs. Compared to hIgG, rIgG was found to confer much better blocking effectiveness for different detection antibodies used to target standard glycoproteins (Fig. 3) and CA125 biomarkers (Fig. 5). This is because of more heterogeneous glycan composition of rIgG than those of human and many other IgGs^42^. Calibration of our microfluidic lectin barcode platform (Fig. 4) yielded very low detection limit for the standard glycoproteins that substantially outperforms that of some highly sensitive lectin-based arrays and sensors reported before, such as the nanoparticle-based bio-bar code platform and the surface plasmon resonance-based sensor^54^,^55^. These results validate our rIgG-based blocking protocol for suppressing non-specific interactions to afford highly sensitive glycoprotein analysis. Because of its glycan heterogeneity, rIgG might provide a broadly applicable blocker for the antibody-lectin assay. While IgG from a single host species was demonstrated for effective blocking, a mixture of different IgGs might be used if necessary.

The feasibility of our system for identifying glycosylation variants was demonstrated via differential glyco-profiling of CA125 originated from three different sources. Cell type and tissue specific variation in glycosylation has been well documented for glycoproteins of biomedical significance, such as disease biomarkers and therapeutic glycoproteins^12^,^41^,^52^. Previous studies have shown different glycosylation patterns for CA125 antigen isolated from human amniotic fluid^45^, placenta^48^, OVCAR3 cell line^44^,^45^, and serum^49^. Wong *et al*. reported the glycans of CA125 from OVCAR3 cells obtained by MS analysis, which mainly consist of bi-, tri- and tetra-antennary bisected complex type N-glycans carrying mostly one fucose and/or one sialic acid, as well as core 1 and 2 type O-glycans with branching core 1 antenna^44^,^45^. Compared to the OVCAR3 cell CA125, serum CA125 was found to have mono-, bi- and tri-antennary complex type N-glycans (mostly core-fucosylated and mono- or di-sialylated), the high-mannose structures, and the core 1 and core 2 type O-glycans^49^. Comparative analysis of OVCAR3 and human amniotic fluid-derived CA125 by Milutinovic *et al*. observed a marked difference in the abundance and structures of *O*-glycans, the multiantennary structures of *N*-glycans, both core- and outer arm-fucosylation, and terminal galactose/GalNAc moieties^45^. Therefore, it is not surprising that we observed notable discrepancy in the CA125 glycan composition among the sources tested in this study and the previous reports. It is noted that this proof-of-concept study did not attempt to comprehensively profile the tissue-specific glyco-patterns of CA125 and thus only 16 lectins were selected to probe representative CA125 glycans that have been studied. Nonetheless, our results should demonstrate the ability of the new method for rapid screening and validation of glycomic fingerprints of diseases.

Taken together with the results for the standard glycoproteins, our approach confers substantially better sensitivity (on the order of 0.1 ng/mL) than that of the recently developed methods that have been improved over the convention lectin arrays, including a sandwich antibody-lectin microarray that uses hIgG as the blocker (on the order of 10 ng/mL)^12^ and the microfluidic lectin array that requires 50 μg/mL pre-labeled glycoproteins^23^. Moreover, the dynamic range for the glycoprotein detection was also substantially expanded. The LOD of CA125 detection is much improved in comparison to the benchtop lectin-detection assay^46^ and comparable with commercial CA125 ELISA kits when some strong-binding lectins, such as SNA, ConA and AAL, were used. Such improvement in analytical performance could be attributed to synergetic effects of microfluidic integration and the highly effective blocking method developed here. Microfluidic transformation leverages the binding kinetics and the sensitivity due to the confined reaction environment and accelerated mass transfer. The blocking strategy is extremely simple yet broadly applicable to substantially reduce the background noise arising from non-specific lectin binding of antibodies. Microfluidic integration of lectin array affords attractive features such as simpler sample pretreatment, much faster analysis and minimal sample requirement. Our system is able to measure low abundant glycoproteins in 20 μL sample, which is much less than the sample volume required for the standard lectin arrays (normally >100 μL)^11^ and the existing microfluidic lectin array (200 μL)^23^. The microfluidic assay normally requires an analysis time of less than three hours. Such abilities are particularly beneficial for biomedical and clinical studies where the availability of large-volume clinical samples is often limited. Therefore, the microfluidic lectin barcode array technology should provide a useful tool that fills the gap in high-throughput glycomic profiling to facilitate deeper understanding of the roles and dynamics of protein glycosylation in disease.

## Methods

### Chemicals and Reagents

Lectins were ordered from Vector Labs (Burlingame, CA) and EY Labs (San Mateo, CA). RNase A and B from bovine pancreas were purchased from Sigma-Aldrich (St. Louis, MO). Human transferrin was from Athens Research & Technology (Athens, GA). Bovine Serum Albumin (IgG-Free, Protease-Free, Jackson Immuno Research Labs) was used as negative control. Biotinylated BSA (Vector Labs) was used as positive control for lectin patterning. Ovarian cancer CA125 proteins purified from different sources were purchased, as listed on Table S2. The CA125 proteins were further purified to remove the sugar additives and preservative using the size exclusion columns (Micro Bio-Spin 30 column, Bio-Rad). The eluted proteins in PBS buffer were measured by the Implen NanoPhotometer™ Pearl (Implen) to determine the concentrations. Carbo-free blocking solution (Vector Labs) and immunoglobulin G (IgG) from human plasma (Athens Research & Technology) and rabbit serum (Sigma-Aldrich) were used as blocking agents. DyLight 488-conjugated streptavidin was obtained from Thermo Scientific (Rockford, IL) and Vector Labs (Burlingame, CA). The antibodies used in this study include rabbit anti-bovine pancreatic ribonuclease polyclonal antibody (Thermo Scientific, Rockford, IL), biotinylated rabbit polyclonal ribonuclease A antibody and rabbit polyclonal transferrin antibody (Novus Biologicals, Littleton, CO), rabbit anti-human transferrin polyclonal antibody (Abcam, Cambridge, MA) and biotinylated mouse anti-human monoclonal CA125 antibody (Clone X52, Fitzgerald, Acton, MA). 3-Glycidoxypropyltrimethoxysilane (GPS, 97%) was purchased from Acros Organics (Morris Plains, NJ). Anhydrous toluene (99.8%) was from Alfa Aesar (Ward Hill, MA). HEPES (BioPerformance Certified, ≥99.5%), tris(hydroxymethyl)aminomethane (≥99.8%) and N,N,N′,N′-tetramethylethylenediamine (TEA) were purchased from Sigma-Aldrich (St. Louis, MO). 1× phosphate-buffered saline solution (1× PBS, Mediatech, Inc.) and sterile water (Mediatech, Inc.) were used as received.

### Microfabrication

The chip fabrication followed a multilayer soft lithography approach^31^. The integrated microfluidic lectin barcode array chip is constructed by a lectin array patterned glass substrate and a two-layer PDMS chip assembly, as illustrated in Fig. 1A. The SU-8 molds on silicon (Si) wafers were fabricated by standard photolithography for the lectin barcode patterning chip, pneumatic layer and microfluidic channel layer, respectively. All the molds were made of SU-8 2050 (MicroChem) with a final thickness of ~30 μm following a procedure as recommended by the manufacturer. The Si molds were pre-treated with trichloro(1H, 1H, 2H, 2H-perfluorooctyl)silane (97%, Sigma-Aldrich, St. Louis, MO) by gas-phase silanization under vacuum for at least 4 hrs. For the lectin barcode patterning chip and the pneumatic layer, PDMS base and curing agent were mixed at an 8:1 ratio (~30 g in total) and poured over mold for the lectin barcode patterning or pneumatic layer, respectively, after being degased. The liquid layer became solidified after baking at 70 °C for about 45 min. Then the PDMS slabs were peeled off from molds and cut into rectangular shape and punched holes for lectin barcode patterning chip preparation or pneumatic control, accordingly. For the sample fluidic layer, the mixture ratio of PDMS was 15:1. Eight grams of the mixture was poured and spin-coated over the mold to form a thin layer of PDMS featuring with the reaction chambers. Each PDMS lectin barcode assay chip was fabricated by aligning a pneumatic layer of PDMS over a sample fluidic layer of PDMS under a stereomicroscope manually and baked at 70 °C overnight in order to integrate the pneumatic layer and the fluidic layer together by forming permanent bonding in between.

### Lectin Barcode Patterning

Epoxy functionalized glass slides were prepared for lectin protein immobilization according to a previous published study^56^ with some modifications. Briefly, plain microscope glass slides (Fisher Scientific) were cleaned with piranha solution, rinsed thoroughly with deionized water, and dried with pure N_2_. Freshly cleaned glass substrates were immerged in 2% GPS in anhydrous toluene with 0.2% TEA under shaking for 1 h at room temperature. The adsorbed excessive silane on GPS-treated glass substrates was removed by rinsing thoroughly with fresh toluene and then isopropanol. GPS-treated substrates were cured at 80 °C for 2 hours to cross-link the monolayer.

The lectin barcode patterning PDMS chip was attached onto a clean GPS-functionalized glass substrate. 15 μL of lectins (0.25–1 mg/mL) dissolved in PBS, PBS with containing the divalent metal ions (i.e., 0.2 mM Ca^2+^, Mg^2+^, and Mn^2+^, unless otherwise noted), or 10 mM HEPES buffer with the divalent metal ions (pH 7.75) were injected through individual microchannels and then incubated at room temperature for 1 to 3 hours or at 4 °C overnight in a humid container. Unbound lectins were washed away by flushing the channels with HEPES buffer with 0.2 mM Ca^2+^, Mg^2+^, and Mn^2+^. The patterning PDMS chip was then peeled off and the patterned slide was washed by water briefly and dried by nitrogen flow. The assembled PDMS chip was cleaned by ethanol, treated with UV Ozone (UVO-Cleaner® 42, Jelight, CA) for 2 min, and finally sealed reversibly onto the patterned glass substrate with the assay microchambers vertically aligned across the lectin array (Fig. 1A). The lectin barcode assay device was first primed with 1X Carbo-free blocking solution with 0.2 mM metal ions for a 30 to 60 min incubation to block unused active epoxy sites on the glass surface as well as non-specific adsorption on PDMS surface. The redundant blocking solution was removed by washing with 10 μL Tris-buffered saline buffer of 20 mM Tris-Cl, 140 mM NaCl, 0.2 mM divalent metal ions, and 0.05% tween 20 (TBSMT, pH 8.05) and the lectin barcode assay device was then ready for use.

### Lectin Barcode Assay

We used 16 lectins (Table S1) to analyze commercially available standard human CA125 purified from three sources: ovarian adenocarcinoma tissue, ovarian carcinoma cell line, and ascetic fluids from ovarian cancer patients (Table S2). The multichannel design allowed us to run three CA125 protein samples and the negative control simultaneously, minimizing the effect of run-to-run variation on discriminating the structural changes. Sample solutions were pumped through each assay microchamber in a stop-flow manner by the integrated pneumatic pumps controlled by a homemade solenoid valve controller via a LabVIEW interface^31^. Using a four-step pumping sequence with the actuation time of 500 ms per step and a pause time of 2–5 seconds, the on-chip pumps were actuated by the opening vacuum at −87 kPa and the closing pressure of 55 kPa to inject the sample at an averaged flow rate of ~0.2 μL/min. Once lectin capture of glycoproteins was completed, the assay chambers were washed with 10 μL of TBSMT buffer and blocked with various blocking agents as specified in the main text. The biotinylated primary antibodies (1 μg/mL) were then injected through and incubated for 15–30 min. After washing with 10 μL of TBSMT, DyLight 488 conjugated streptavidin (1 μg/mL) was flowed into the lectin barcode array chip for 10 min. After thoroughly rinsing with 10 μL of TBSMT, the chip was imaged under a Zeiss Axiovert A1 inverted fluorescence microscope equipped with an AxioCam MRm CCD camera and a LED excitation light source (Thorlabs, Newton, NJ). Acquired fluorescence images were analyzed using ImageJ (NIH, http://rsbweb.nih.gov/ij/) to quantify the signal intensity. The mean intensity measured for each barcode spot was subtracted by averaged local backgrounds and the data were used without normalization^11^.

## Additional Information

**How to cite this article**: Shang, Y. *et al*. Integrated Microfluidic Lectin Barcode Platform for High-Performance Focused Glycomic Profiling. *Sci. Rep*. **6**, 20297; doi: 10.1038/srep20297 (2016).

## Supplementary Material

Supplementary Information
