# Supplementary material for: Integrated Microfluidic Lectin Barcode Platform for High-Performance Focused Glycomic Profiling
Source: Sci Rep. 2016 Feb 2;6:20297. doi: 10.1038/srep20297 (PMC4735825; doi:10.1038/srep20297)
Supplement: Supplementary Information [file srep20297-s1.pdf]

# Integrated Microfluidic Lectin Barcode Platform for High-Performance Focused Glycomic Profiling

Yuqin Shang[1], Yun Zeng[2], and Yong Zeng[1,3*]

1. Department of Chemistry, University of Kansas, Lawrence, KS 66045;
2. College of Water Resource and Hydropower, Sichuan Agricultural University, Ya'an, Sichuan 625014, P.R. China
3. University of Kansas Cancer Center, Kansas City, KS 66160
* Corresponding author.
Tel: (785) 864-8105; Fax: (785) 864-5396; E-mail: yongz@ku.edu

Table S1. List of Lectins used in this work and their glycan binding specificities.

| | **Lectins** | **Origin** | **Binding specificity** |
|---|---|---|---|
| **1** | PNA | *Arachis hypogaea* | Gal β1-3 GalNAcα-Thr/Ser (T antigen) |
| **2** | LCA | *Lens culinaris* | Fucα1-6GlcNAc and α-Man, α-Glc |
| **3** | SBA | *Glycine max* | Terminal GalNAc (especially GalNAcα1-3Gal) |
| **4** | AAL | *Aleuria aurantia* | Terminal αFuc and ±Sia-$Le^x$ |
| **5** | MAL II | *Maackia amurensis II* | Siaα 2-3Gal |
| **6** | SNA | *Sambucus nigra* | Siaα2-6Gal/GalNAc |
| **7** | PHA-L | *Phaseolus vulgaris* | Tri- and tetra-antennary complex oligosaccharides |
| **8** | ECA | *Erythrina cristagalli* | Lac/LacNAc |
| **9** | RCA120 | *Ricinus communis* | Galactose, Lac/LacNAc |
| **10** | DSL | *Datura stramonium* | $(GlcNAc)_n$, polyLacNAc and LacNAc (NA3, NA4) |
| **11** | GSL-II | *Griffonia simplicifolia* | Agalactosylated N-glycan |
| **12** | ConA | *Canavalia ensiformis* | α-Man (inhibited by presence of bisecting GlcNAc), α-Glc, complex-type *N*-glycans |
| **13** | GNA | *Galanthus nivalis* | non-substituted α1-3 and α1-6 Man |
| **14** | UEA-I | *Ulex europaeus* | Fucα1-2LacNAc |
| **15** | VVL | *Vicia villosa* | α-, β-linked terminal GalNAc and GalNAcα-Thr/Ser (Tn) |
| **16** | WGA | *Triticum unlgaris* | GlcNAcβ1-4GlcNAcβ1-4GlcNAc, β-GlcNAc and multivalent Sia |

Table S2. Datasheet of three commercial CA125 protein samples used in this work

| **Vendor** | **MeridianLifeScience** | **MeridianLifeScience** | **Fitzgerald Industries** |
|---|---|---|---|
| Catalog No. | A86928H | A97180H | 30-AC21 |
| Source | Human ovarian adenocarcinoma tissue | Ovarian carcinoma cell line | Ascitic fluids from ovarian cancer patients |
| Purity/ Purification | >50% pure by SDS-PAGE | Known Contaminants: CA19-9= 124 Units/mL(<0.1%) CA15-3= 2.15 Units/mL(<0.1%) | >95% pure |
| Concentration | 650,000 Units/mL | 83,840 Units/mL | 750,000 Units/mL |
| Format & Buffer | Liquid in PBS (pH7.4) with 3% sucrose and 0.05% NaN3 | Liquid in PBS (pH7.4 ± 0.2) with 0.1% NaN3 | Liquid in PBS (pH7.2) with 3% sucrose and 0.05% NaN3 |

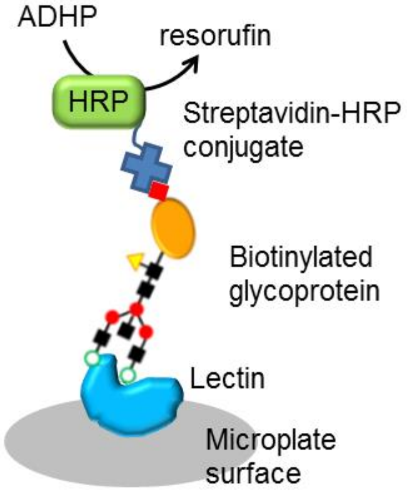


**Figure S1.** Scheme of the microplate-based lectin assay using horseradish peroxidase-conjugated streptavidin and the QuantaRed HRP substrate (ADHP) for absorbance or enhanced chemifluorescent detection.

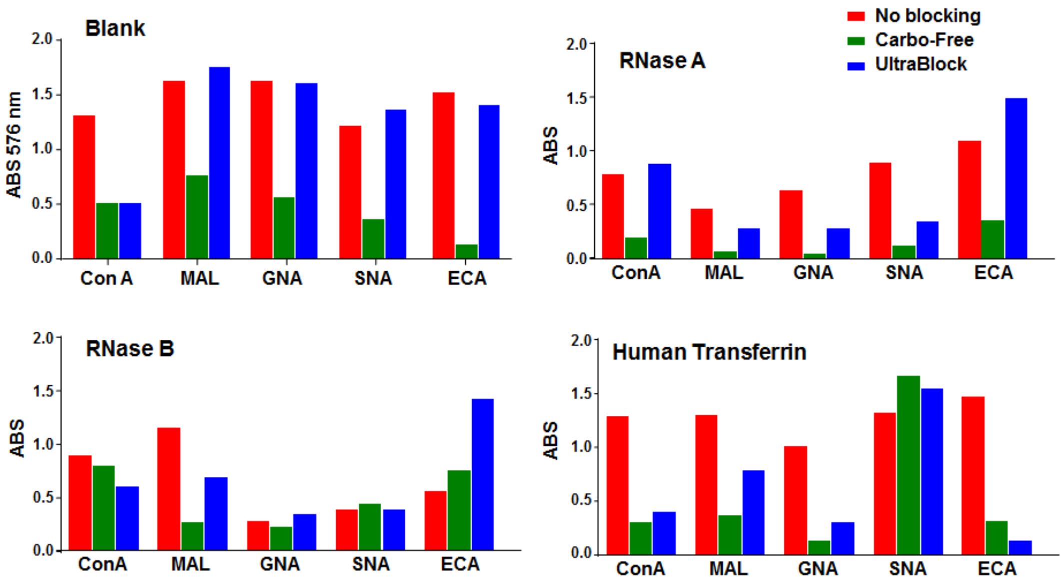


**Figure S2.** Effects of different blocking solutions, Carbo-Free (Vector) and UltraBlock (AbD Serotec), on the background and binding of two biotinylated glycoproteins and one control protein (1 μM) using six lectins deposited in a 384-well microtiter plate.

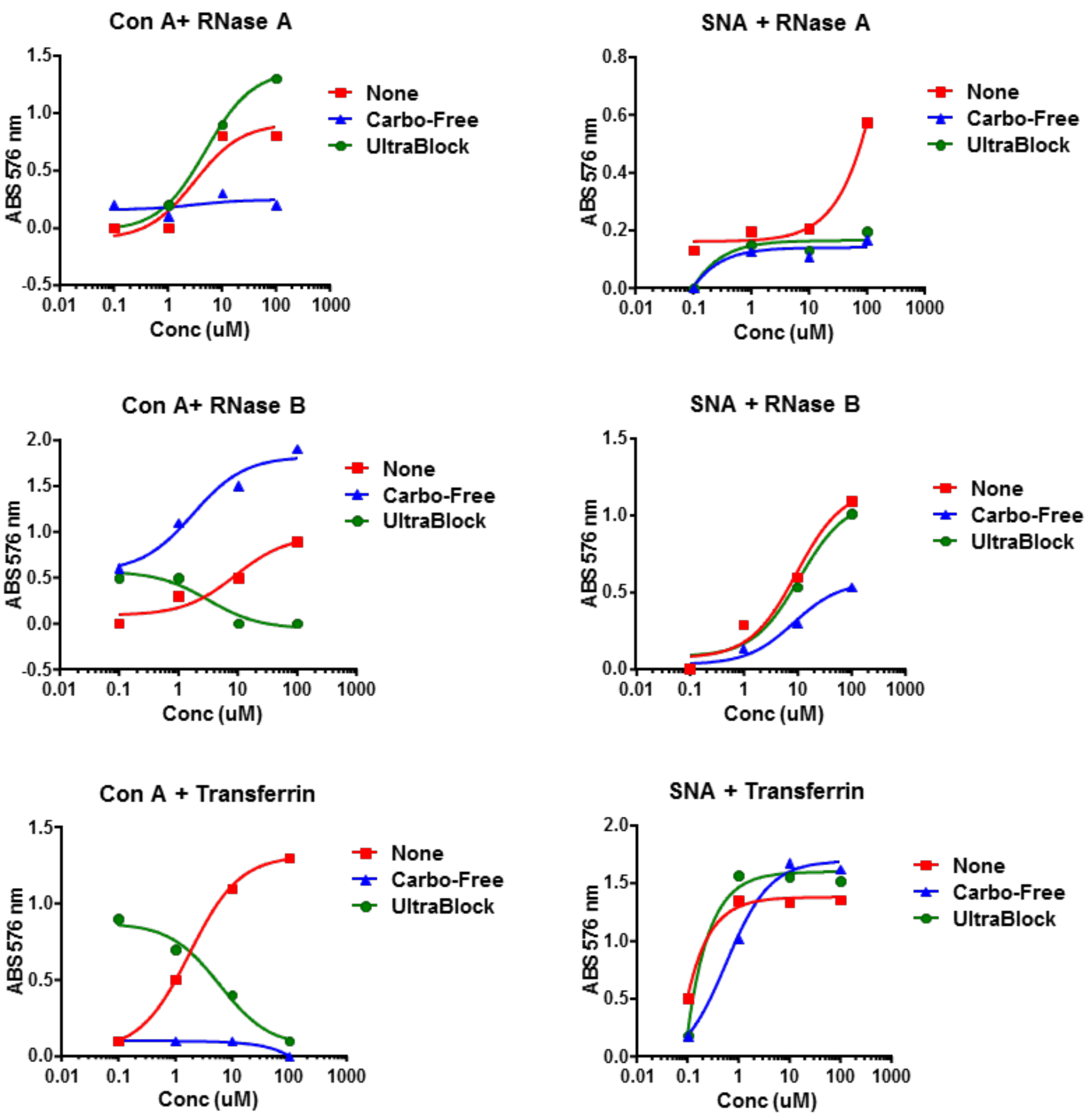


**Figure S3**. Concentration titration of the standard protein/glycoproteins using Con A and SNA confirms the blocking effectiveness of the Carbo-Free solution and suggests no inhibition on lectin-glycan binding, in contrast to the UltraBlock solution.

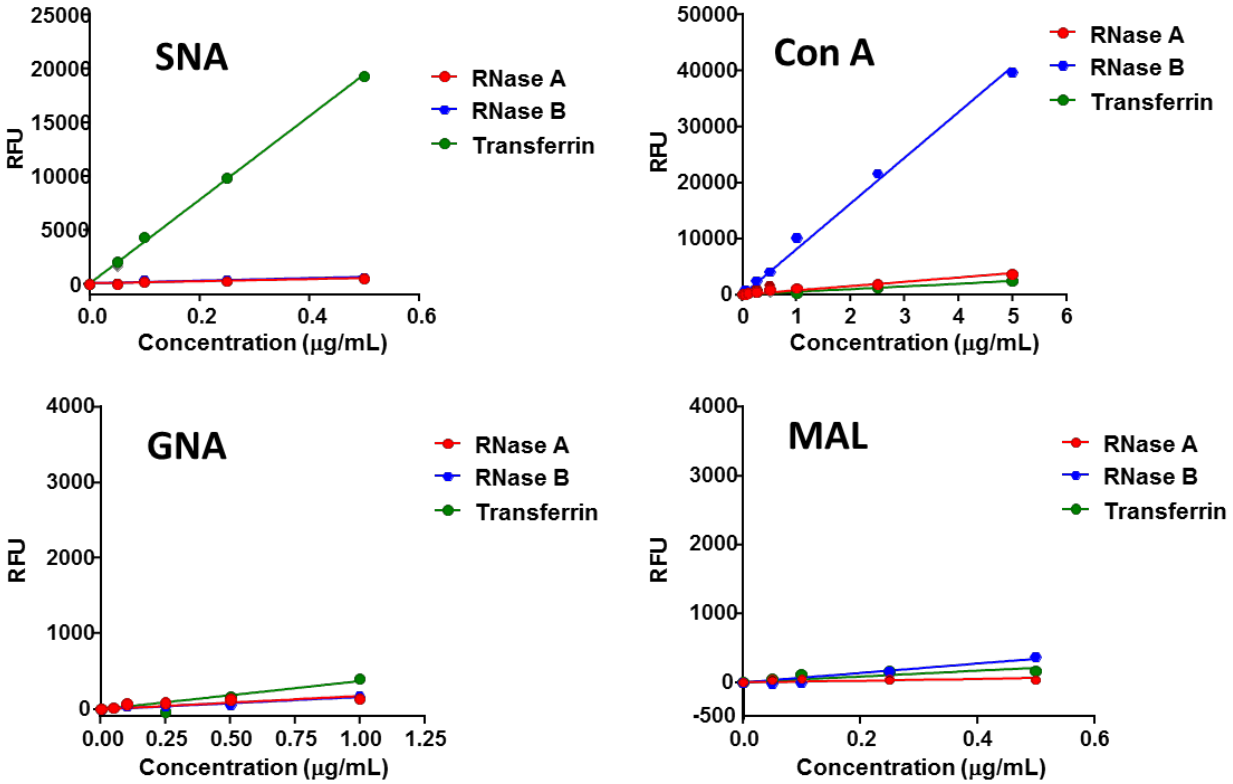


**Figure S4.** Quantitative detection of three standard protein and glycoproteins using various lectins. Assay conditions: buffer, TBST (20 mM Tris (pH 7.6), 150 mM NaCl, and 0.05% Tween 20), blocking buffer, Carbo-Free; HRP-STV, 1.5 mg/ml; overnight binding; QuantaRed fluorescence detection.

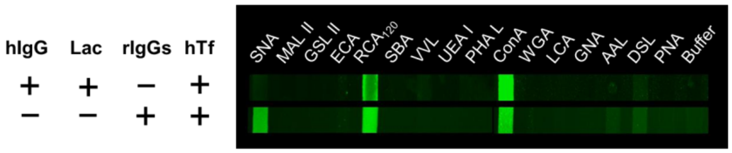


**Figure S5.** Comparison of blocking performance of a mixture of hIgG and lactose (top) and rIgG (bottom) for glycan profiling of hTf. The hIgG and lactose mixture blocker inhibits specific glycan binding for SNA and AAL.
